# Supplementary material for: Live-cell single-molecule tracking highlights requirements for stable Smc5/6 chromatin association in vivo
Source: eLife. 2021 Apr 16;10:e68579. doi: 10.7554/eLife.68579 (PMC8075580; doi:10.7554/eLife.68579)
Supplement: Supplementary file 1. — Data from individual experimental repeats of ura4 loss assay in Figure 4C. [file elife-68579-supp1.docx]

| **Strain** | **Rate of *ura4* loss per cell per generation** | | |
| --- | --- | --- | --- |
| **Experiment 1** | **Off**  **(+thiamine)** | **On**  **(-thiamine)** | **Relative**  **to Off** |
| *smc6*^+^ | 2.2 x 10^-8^ | 4.6 x 10^-8^ | 2.09 |
| *smc6*-74 (A151T) | 1.4 x 10^-7^ | 3.83 x 10^-7^ | 2.71 |
| *smc6*-X (R706C) | 8.4 x 10^-9^ | 1.6 x 10^-6^ | 194.00 |
| *nse3-R254E* | 6.0 x 10^-8^ | 2.4 x 10^-7^ | 3.99 |
|  |  | | |
| **Experiment 2** |  | | |
| *smc6*^+^ | 3.2 x 10^-8^ | 5.7 x 10^-8^ | 1.77 |
| *smc6*-74 (A151T) | 2.3 x 10^-7^ | 1.56 x 10^-6^ | 6.78 |
| *smc6*-X (R706C) | 1.3 x 10^-7^ | 7.7 x 10^-6^ | 61.2 |
| *nse3-R254E* | 7.2 x 10^-8^ | 6.0 x 10^-7^ | 8.36 |
|  |  | | |
| **Experiment 3** |  | | |
| *smc6*^+^ | 1.6 x 10^-8^ | 1.8 x 10^-8^ | 1.12 |
| *smc6*-74 (A151T) | 7.09 x 10^-8^ | 3.98 x 10^-7^ | 5.61 |
| *smc6*-X (R706C) | 1.3 x 10^-7^ | 1.4 x 10^-5^ | 105.70 |
| *nse3-R254E* | 1.3 x 10^-8^ | 4.1 x 10^-7^ | 32.20 |
