# Supplementary material for: Live-cell single-molecule tracking highlights requirements for stable Smc5/6 chromatin association in vivo
Source: eLife. 2021 Apr 16;10:e68579. doi: 10.7554/eLife.68579 (PMC8075580; doi:10.7554/eLife.68579)
Supplement: Supplementary file 2. — Strains used during this study. [file elife-68579-supp2.docx]

| **Strain No.** | **Genotype** | **Reference** |
| --- | --- | --- |
| TJE323 | *loxP-nse4-mEos3.2-loxM3 ura4-D18 leu1-32 ade6-704* | This study |
| TJE350 | *loxP-smc6-mEos3.2-loxM3 ura4-D18 leu1-32 ade6-704* | This study |
| TJE496 | *smc1-loxP-mEos3.2:kanMX6-loxM3 ura4-D18 leu1-32* | This study |
| TJE480 | *loxP-nse4-mEos3.2-loxM3 loxP-smc6-T135V-loxM3 ura4-D18 leu1-32 ade6-704* | This study |
| TJE477 | *loxP-nse4-mEos3.2-loxM3 loxP-smc6-T135L-loxM3 ura4-D18 leu1-32 ade6-704* | This study |
| TJE475 | *loxP-nse4-mEos3.2-loxM3 loxP-smc6-T135F-loxM3 ura4-D18 leu1-32 ade6-704* | This study |
| TJE410 | *loxP-nse4-mEos3.2-loxM3 smc6-A151T ura4-D18 leu1-32 ade6-704* | This study |
| TJE719 | *loxP-nse4-mEos3.2-loxM3 loxP-smc6-R150A-loxM3 ura4-D18 leu1-32 ade6-704* | This study |
| TJE711 | *loxP-nse4-mEos3.2-loxM3 loxP-smc5-R77A-loxM3 ura4-D18 leu1-32 ade6-704* | This study |
| TJE509 | *loxP-nse4-mEos3.2-loxM3 smc6-F528A ura4-D18 leu1-32 ade6-704* | This study |
| TJE483 | *loxP-nse4-mEos3.2-loxM3 smc5-R609E R615E ura4-D18 leu1-32 ade6-704* | This study |
| TJE671 | *loxP-nse4-mEos3.2-loxM3 smc5-Y612G ura4-D18 leu1-32 ade6-704* | This study |
| TJE418 | *loxP-nse4-mEos3.2-loxM3 smc6-R706C ura4-D18 leu1-32 ade6-704* | This study |
| TJE492 | *loxP-nse4-mEos3.2-loxM3 loxP-nse3-R254E-loxM3 ura4-D18 leu1-32 ade6-704* | This study |
| TJE730 | *loxP-nse4-mEos3.2-loxM3 brc1::hphMX6 ura4-D18 leu1-32 ade6-704* | This study |
| TJE734 | *loxP-nse4-mEos3.2-loxM3 nse6::kanMX6 ura4-D18 leu1-32 ade6-704* | This study |
| TJE796 | *nse6-loxP-mEos3.2-loxM3 ura4-D18 leu1-32* | This study |
| TJE816 | *loxP-nse4-mEos3.2-loxM3 hta1-S129A:ura4 hta2-S128A:his3 his3-D1 ura4-D18 leu1-32* | This study |
| TJE393 | *rad21-loxP-mEos3.2:kanMX6-loxM3 ura4-D18 leu1-32* | This study |
| TJE586 | *nse2-loxP-mEos3.2:kanMX6-loxM3 ura4-D18 leu1-32* | This study |
| TJE522 | *cnd2-loxP-mEos3.2:kanMX6-loxM3 ura4-D18 leu1-32* | This study |
| TJE886 | *loxP-nse4-mEos3.2-loxM3 mcm4-loxP-yEGFP:KanMX6-loxM3 ura4-D18 leu1-32 ade6-704* | This study |
| THE 884 | *loxP-nse4-mEos3.2-loxM3 loxP-brc1-T672A-loxM3 ura4-D18 leu1-32* | This study |
| TJE828 | *nse6-loxP-mEos3.2-loxM3 smc6-74 ura4-D18 leu1-32* | This study |
| TJE830 | *nse6-loxP-mEos3.2-loxM3 smc6-X ura4-D18 leu1-32* | This study |
| TJE888 | *loxP-nse4-mEos3.2-loxM3 brc1::hphMX6 nse6::kanMX6 ura4-D18 leu1-32 ade6-704* | This study |
| HQD87 | *loxP-smc5+-ura4-loxM3 ura4-D18 leu1-32 ade6-704* | This study |
| DE297 | *loxP-smc6+-ura4-loxM3 ura4-D18 leu1-32 ade6-704* | This study |
| DE285 | *loxP-smc6-T135V-loxM3 ura4-D18 leu1-32 ade6-704* | This study |
| DE283 | *loxP-smc6-T135L-loxM3 ura4-D18 leu1-32 ade6-704* | This study |
| DE281 | *loxP-smc6-T135F-loxM3 ura4-D18 leu1-32 ade6-704* | This study |
| DE279 | *loxP-smc6-R150A-loxM3 ura4-D18 leu1-32 ade6-704* | This study |
| DE342 | *loxP-smc5-R77A-loxM3 ura4-D18 leu1-32 ade6-704* | This study |
| DE273 | *smc6-A151T ura4-D18 leu1-32 ade6-704* | Lab strain |
| JMM1188 | *ura4-D18 leu1-32 ade6-704* | Lab strain |
| JMM1162 | *nmt41:rtf1:sup35 RTS1-ura4-RTS1 ade6-704 leu1-32* | *Lambert et al 2005* |
| JMM1171 | *rhp51::NAT nmt41:rtf1:sup35 RTS1-ura4-RTS1 ade6-704 leu1-32* | *Lambert et al 2005* |
| JMM1371 | *smc6-A151T nmt41:rtf1:sup35 RTS1-ura4-RTS1 ade6-704 leu1-32* | This study |
| JMM1375 | *smc6-R706C nmt41:rtf1:sup35 RTS1-ura4-RTS1 ade6-704 leu1-32* | This study |
| DE331 | *nse3-R254E nmt41:rtf1:sup35 RTS1-ura4-RTS1 ade6-704 leu1-32* | This study |
